# The clinical benefit of mepolizumab replacing omalizumab in uncontrolled severe eosinophilic asthma

**DOI:** 10.1111/all.13850

**Published:** 2019-07-01

**Authors:** Kenneth R. Chapman, Frank C. Albers, Bradley Chipps, Xavier Muñoz, Gilles Devouassoux, Miguel Bergna, Dmitry Galkin, Jay Azmi, Dalal Mouneimne, Robert G. Price, Mark C. Liu

**Affiliations:** ^1^ Asthma and Airway Centre University Health Network, University of Toronto Toronto Ontario Canada; ^2^ Global Respiratory Medical Franchise GSK Research Triangle Park North Carolina; ^3^ Capital Allergy and Respiratory Disease Center Sacramento California; ^4^ Pulmonology Department Hospital Universitari Vall d'Hebron Barcelona Spain; ^5^ Ciber Enfermedades Respiratorias Madrid Spain; ^6^ Service de Pneumologie, Hôpital de la Croix Rousse, Hospices Civils de Lyon UCB Lyon 1 Lyon France; ^7^ Respiratory Research CEMER, Vicente Lopez Buenos Aires Argentina; ^8^ Respiratory TAU GSK Uxbridge UK; ^9^ Global Medical Affairs GSK House Brentford Middlesex UK; ^10^ Clinical Statistics GSK Stevenage UK; ^11^ Divisions of Allergy and Clinical Immunology, Pulmonary and Critical Care Medicine Johns Hopkins Asthma and Allergy Center Baltimore Maryland

**Keywords:** ACQ‐5, asthma control, mepolizumab, omalizumab, severe eosinophilic asthma

## Abstract

**Background:**

Mepolizumab and omalizumab are treatments for distinct but overlapping severe asthma phenotypes.

**Objective:**

To assess if patients eligible for both biologics but not optimally controlled with omalizumab experience improved asthma control when switched directly to mepolizumab.

**Methods:**

OSMO was a multicenter, open‐label, single‐arm, 32‐week trial in patients with ≥2 asthma exacerbations in the year prior to enrollment, despite receiving high‐dose inhaled corticosteroids and other controller(s), plus omalizumab (≥4 months). At baseline, patients with blood eosinophil counts ≥150 cells/µL (or ≥300 cells/µL in the prior year) and an Asthma Control Questionnaire (ACQ)‐5 score ≥1.5 discontinued omalizumab and immediately commenced mepolizumab 100 mg subcutaneously every 4 weeks. Endpoints included change from baseline in ACQ‐5 score (primary), St George's Respiratory Questionnaire (SGRQ) score and the proportions of ACQ‐5 and SGRQ responders, all at Week 32, and the annualized exacerbation rate over the study period.

**Results:**

At Week 32 (intent‐to‐treat population [n = 145]), the least squares (LS) mean changes (standard error [SE]) in ACQ‐5 and SGRQ total scores were −1.45 (0.107) and −19.0 (1.64) points; with 77% and 79% of patients achieving the minimum clinically important differences (ACQ‐5: ≥0.5 points; SGRQ: ≥4 points), respectively. The annualized rate of clinically significant exacerbations was 1.18 events/year, a 64% reduction from 3.26 events/year during the previous year. Safety and immunogenicity profiles were consistent with previous trials.

**Conclusion:**

After directly switching from omalizumab to mepolizumab, patients with uncontrolled severe eosinophilic asthma experienced clinically significant improvements in asthma control, health status, and exacerbation rate, with no tolerability issues reported.

## Graphical abstract

Minimal clinically important difference for improvement in asthma control questionnaire‐5 and St. George's Respiratory Questionnaire total scores was achieved by 77% and 79% of patients, respectively. Annualized rate of clinically significant exacerbations was reduced from 3.26 to 1.18 events/year. Safety and immunogenicity profiles of mepolizumab were consistent with previous placebo‐controlled trials in severe eosinophilic asthma.

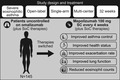

**Abbreviations**
ACQ‐5: Asthma Control Questionnaire‐5
ADA: anti‐drug antibody
AE: adverse event
AESI: adverse event of special interest
BMI: body mass index
CI: confidence interval
ECG: electrocardiogram
ER: emergency room
FeNO: fractional exhaled nitric oxide
FEV_1_: forced expiratory volume in 1 second
FVC: forced vital capacity
GEE: generalized estimating equation
GINA: Global Initiative for Asthma
ICS: inhaled corticosteroid
IgE: immunoglobulin E
IL: interleukin
ITT: intent‐to‐treat
LABA: long‐acting β_2_‐agonist
LS: least squares
LTRA: leukotriene receptor antagonist
MCID: minimum clinically important difference
OCS: oral corticosteroid
SAE: severe adverse event
SC: subcutaneous
SCS: systemic corticosteroids
SD: standard deviation
SE: standard error
SGRQ: St George’s Respiratory Questionnaire
TSQM‐9: Treatment Satisfaction Questionnaire for Medication‐9

## 1 INTRODUCTION

Asthma is a heterogenous condition that affects approximately 235 million people worldwide.^1^ Although most patients with asthma are able to manage their symptoms and enjoy a good quality of life, 5%‐10% of patients suffer from severe asthma.^2^ Severe asthma is associated with significant morbidity and mortality,^3^ and accounts for approximately 50% of asthma care costs.^4^ Patients with severe asthma typically require regular treatment with high‐dose inhaled corticosteroids (ICS), plus an additional controller or systemic corticosteroids (SCS) to prevent their disease from becoming uncontrolled.^2^ Despite this therapy, a subset of patients continue to have uncontrolled disease.

Severe asthma comprises different phenotypes driven by distinct pathophysiological processes.^5^ However, some severe asthma phenotypes overlap in terms of clinical and physiological characteristics, biomarker expression, and treatment response.^2^, ^5^ In clinical practice, severe allergic asthma and severe eosinophilic asthma are recognized as distinct, but potentially overlapping phenotypes of severe asthma.^6^ Severe allergic asthma is characterized by an early age of onset, high levels of serum immunoglobulin E (IgE), high fractional exhaled nitric oxide (FeNO), clinically relevant sensitization to common aeroallergens and eosinophilic inflammation; severe eosinophilic asthma is characterized by a later age of onset, peripheral eosinophilia, high FeNO, and frequent exacerbations.^2^, ^5^ Due to the unmet clinical need within these severe asthma populations, novel biologic therapies that target the immunologic mediators of disease have been developed.^4^

Omalizumab is an anti‐IgE antibody indicated for use in patients with moderate‐to‐severe allergic asthma.^7^ The humanized monoclonal antibody binds to the FcεRI binding domain of free circulating IgE, inhibits binding of IgE to its receptors, and decreases free IgE levels in serum.^8^ In patients with severe asthma, omalizumab treatment decreases exacerbations, improves asthma control and improves patient quality of life.^9^, ^10^, ^11^ Omalizumab is recommended by the Global Initiative for Asthma (GINA) as a potential Step 5 treatment for patients with severe allergic asthma.^12^ However, in some patients, symptoms remain uncontrolled despite omalizumab therapy. The European Respiratory Society/American Thoracic Society guidelines note that if symptoms do not improve within 4 months of initiating omalizumab treatment, further administration is unlikely to be beneficial.^2^

Mepolizumab is an anti‐interleukin (IL)‐5 humanized mono‐clonal antibody indicated for use in severe eosinophilic asthma.^13^ By binding with high affinity to free IL‐5, mepolizumab blocks the interaction between IL‐5 and the eosinophil cell surface receptor IL5Rα, preventing IL‐5‐driven eosinophil proliferation, survival and differentiation.^14^ Mepolizumab effectively decreases peripheral blood eosinophil counts and exacerbations,^15^, ^16^, ^17^ reduces oral glucocorticoid dependence,^15^ and improves lung function and health‐related quality of life in patients with severe eosinophilic asthma.^16^, ^18^ Mepolizumab is also recommended by GINA as a potential Step 5 treatment for patients with severe eosinophilic asthma.^12^

Omalizumab has been available for clinical use since 2003.^7^ As such, some patients with severe asthma who are eligible for both biologics have been receiving omalizumab.^18^, ^19^ The primary objective of this study was to identify patients with severe eosinophilic asthma being treated with omalizumab whose disease was not optimally controlled, and to evaluate, in a pragmatic setting, any improvement in asthma control following a switch from omalizumab to mepolizumab without a washout period.

## 2 METHODS

### 2.1 Study design and treatment

OSMO (Omalizumab Switch to MepOlizumab study) was an open‐label, single‐arm, multicenter trial in patients with severe eosinophilic asthma not optimally controlled by omalizumab treatment (NCT02654145). Details of study locations are provided in the Appendix S1. The single‐arm study design was chosen in order to focus on the switch from omalizumab to mepolizumab in a manner that reflects clinical practice. Following a prescreening phase, which occurred over a 2‐week period, patients attended a screening visit (Visit 1) to assess eligibility for the study. Eligible patients entered a 1‐4 weeks run‐in period, during which their continued eligibility was assessed. All maintenance therapy, including omalizumab, was continued throughout the run‐in period. At the baseline study visit (Visit 2), patients discontinued omalizumab treatment and switched to mepolizumab 100 mg subcutaneously every 4 weeks for 32 weeks (final dose Week 28). With the exception of omalizumab, patients continued their maintenance therapies in unchanged dosages throughout the study period.

### 2.2 Patients

Eligible patients were ≥12 years of age (or ≥18 years of age where local regulations restricted enrollment to adults), had a physician's diagnosis of asthma for ≥2 years according to National Heart, Lung and Blood Institute or GINA guidelines,^20^, ^21^ and a peripheral blood eosinophil count ≥ 150 cells/µL at Visit 1, or ≥300 cells/µL in the 12 months prior to Visit 1. All patients had documented requirement for high‐dose ICS for the 12 months prior to Visit 1, plus an additional controller (long‐acting β_2_ agonist [LABA], leukotriene receptor antagonist [LTRA], long‐acting anticholinergic, or theophylline) with or without maintenance oral corticosteroids (OCS). Patients were considered to have suboptimal control on omalizumab if they had an Asthma Control Questionnaire‐5 (ACQ‐5) score of ≥1.5 at Visits 1 and 2 and a history of ≥2 exacerbations requiring treatment with SCS (intramuscular, intravenous, or oral) in the 12 months prior to Visit 1, despite treatment with omalizumab for at least 4 months. For patients receiving omalizumab for ≥8 months, at least 1 exacerbation must have occurred on omalizumab treatment. Patients were excluded if, in the opinion of the investigator, omalizumab treatment had provided significant clinical benefit in the past 12 months, despite the patient experiencing ≥2 exacerbations. Further inclusion and exclusion criteria are described in the Appendix S1. This study was conducted in accordance with International Conference for Harmonization Good Clinical Practice, applicable country‐specific requirements and ethical principles outlined in the Declaration of Helsinki. All patients provided written informed consent prior to any study‐related activities. The study was approved by local ethics review boards of the participating sites.

### 2.3 Endpoints and assessments

The primary endpoint was mean change from baseline at Week 32 in ACQ‐5 score. The ACQ‐5 score has a range of 0‐6 with higher scores indicating worse asthma control. The minimum clinically important difference (MCID) in ACQ‐5 score has been established as 0.5 points.^22^ Secondary endpoints were mean change from baseline at Week 32 in St George's Respiratory Questionnaire (SGRQ) score, frequency of clinically significant asthma exacerbations over the 32‐week study period (Appendix S1), and ratio to baseline at Week 32 of blood eosinophil count. SGRQ scores range from 0 to 100, with higher scores indicating worse health status (MCID = 4‐point change in score).^23^

Additional endpoints included the percentage of patients achieving ≥0.5‐point reduction from baseline in ACQ‐5 score, the percentage of patients achieving ≥4‐point reduction from baseline in SGRQ total score, and mean change from baseline in pre‐ and postbronchodilator FEV_1_, all at Week 32. We also assessed the frequency of exacerbations requiring an emergency room (ER) visit or hospitalization over the 32‐week study period, patient‐ and clinician‐rated response to therapy, and the mean change from baseline in Treatment Satisfaction Questionnaire for Medication (TSQM‐9). The TSQM‐9 Overall Satisfaction Scale score has a range of 0‐100, with higher scores indicating greater satisfaction. Levels of inflammatory biomarkers were also assessed (Appendix S1).

Safety endpoints were the frequency of adverse events (AEs), serious AEs (SAEs) and AEs of special interest (AESIs; Appendix S1). AEs were recorded on a worksheet by patients and documented by study staff at each visit. Immunogenicity endpoints included the presence of anti‐drug antibodies (ADAs), defined as any antibody isotype directed against mepolizumab; binding assays were performed at baseline and Weeks 12, 28, and 32. Samples testing positive for ADAs were further tested for the presence of neutralizing antibodies.

### 2.4 Statistical analysis

The intent‐to‐treat (ITT) population, defined as all patients who were enrolled in the study and received ≥1 dose of mepolizumab, formed the primary analysis population. To take into account a possible “placebo effect” or “Hawthorne effect” due to clinical trial participation, the primary endpoint data were also compared to two “historical placebo” estimates produced from meta‐analyses of previous studies of mepolizumab in patients with severe eosinophilic asthma. The first, a meta‐analysis of DREAM (NCT01000506)^17^ and MENSA (NCT01691521)^16^ using all placebo patients, estimated a “placebo effect” of −0.55 mean change from baseline in ACQ‐5 score (standard error [SE]: 0.05); and the second, a meta‐analysis of MENSA and MUSCA (NCT02281318)^18^ using placebo patients previously treated with omalizumab, estimated a “placebo effect” of −0.11 mean change from baseline in ACQ‐5 score (SE: 0.14).

We estimated that a sample size of 120 would provide 90% power to declare statistical significance over the historical “placebo effect” of −0.55 mean improvement from baseline in ACQ‐5 score at a two‐sided significance level of 5%. These estimates were made based on a residual standard deviation (SD) of 0.96 and assumed 15% of patients would withdraw from the study prematurely. Comparisons in all efficacy endpoints were made back to baseline. Questionnaires, blood eosinophil counts, and lung function tests were analyzed using a mixed model repeated measures model (MMRM) with covariates of region, baseline maintenance OCS therapy (OCS, no OCS), exacerbations in the prior year, and visit. For ACQ‐5, the primary comparison was based on estimates from week 32. Exacerbation data were analyzed using a generalized estimating equation (GEE) model 24 assuming a negative binomial distribution with a covariate of treatment period (pretreatment, 32 weeks study period). For blood eosinophil counts, where a result of zero was recorded, a small value (ie, half the minimum nonzero result) was imputed prior to log‐transformation.

Post hoc subgroup analyses were additionally conducted in patients who were or were not receiving maintenance OCS at baseline, and in patients who experienced ≥2 exacerbations during the on‐treatment period. All statistical analyses were performed using SAS version 9.4 (SAS Institute).

## 3 RESULTS

### 3.1 Patient population

**Figure 1:**
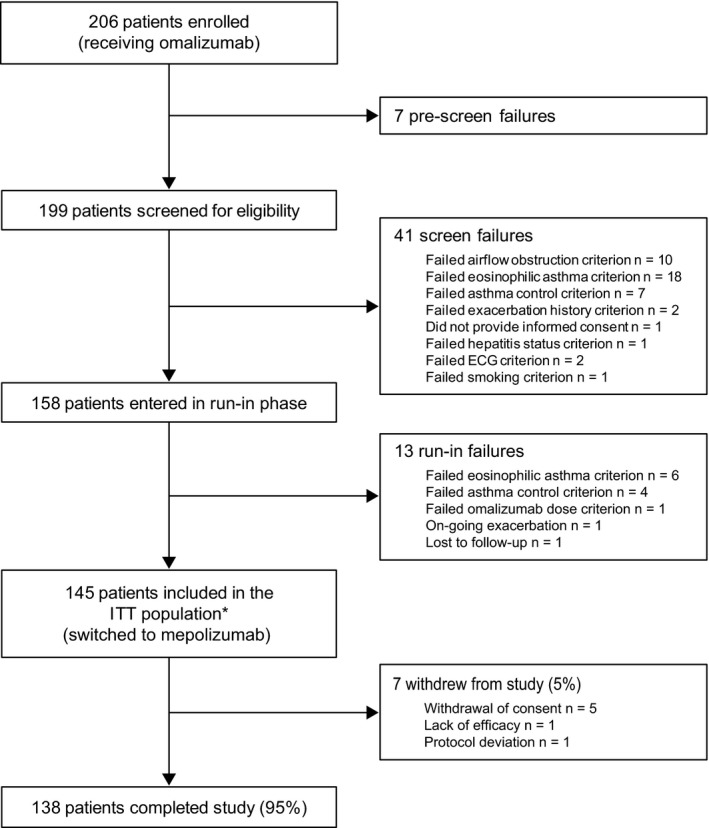
Overview of study design and patient flow. One patient failed two inclusion/exclusion criteria at screening *Fourteen patients (10%) received mepolizumab and were included in the ITT population despite having failed ≥ 1 eligibility criterion, see Table S1 for details. There were two patients that discontinued treatment with mepolizumab due to adverse events (urticaria and ECG QT prolonged) but who were not withdrawn and completed the study. ECG, electrocardiogram; ITT, intent‐to‐treat

The study was conducted from March 17, 2016, to May 31, 2017. Overall, 206 patients currently receiving omalizumab were enrolled in the study, of whom 145 were switched to mepolizumab and were included in the ITT population. Seven patients (5%) withdrew from the study and two additional patients (1%) withdrew from the investigational product (mepolizumab) due to AEs but completed the study (Figure 1). Demographic and baseline clinical characteristics are summarized in Table 1. Patients in the ITT population had an average age of 53.6 years and 59% were women. Overall, 52% and 48% of patients previously received omalizumab every 2 weeks and every 4 weeks, respectively, with a median (range) prior omalizumab treatment duration of 29.6 (4, 161) months (Table 1).

**Table 1:** Summary of demographic and baseline clinical characteristics (ITT population)

Characteristic	Mepolizumab 100 mg SC (N = 145)
Age, years	
12‐17, n (%)	2 (1)
18‐64, n (%)	112 (77)
≥65, n (%)	31 (21)
Mean (SD)	53.6 (13.83)
Gender, female, n (%)	86 (59)
Race, n (%)	
White	128 (88)
Asian	5 (3)
Black or African American	11 (8)
Mixed	1 (<1)
Ethnicity, n (%)	
Non‐Hispanic/Latino	107 (74)
Body mass index, kg/m^2^, mean (SD)	30.2 (6.27)
Duration of asthma, years, mean (SD)	25.6 (16.81)
Allergy comorbidities	
Allergic rhinitis	29 (20)
Nasal polyps	20 (14)
Maintenance OCS use at baseline, n (%)	35 (24)
Median (range) dose, mg/day prednisolone equivalent	10 (4, 40)
Concurrent therapy use at baseline, n (%)	
ICS^a^	145 (100)
Mean (SD) dose, mcg/day fluticasone propionate (DPI) equivalent	997 (574)
LABA^b^	145 (100)
SABA	125 (86)
LTRA	70 (48)
LAMA	62 (43)
Xanthine	14 (10)
Exacerbations^c^ in the past 12 mo	
Clinically significant exacerbations, mean (SD)	3.3 (2.65)
Exacerbations requiring ER/hospitalization, n (%)	43 (30)
Exacerbations requiring hospitalization, n (%)	17 (12)
Spirometry assessments at baseline, mean (SD)	
Prebronchodilator % of predicted FEV_1_	59.5 (17.94)
Prebronchodilator FEV_1_, L	1.76 (0.68)
Postbronchodilator FEV_1_, L^d^	1.99 (0.80)
Prebronchodilator FEV_1_/FVC	0.62 (0.13)
Postbronchodilator FEV_1_/FVC^d^	0.65 (0.13)
FEV_1_ reversibility, L	0.22 (0.26)
Baseline ACQ‐5 score^e^, mean (SD)	3.19 (0.937)
Baseline SGRQ total score^f^, mean (SD)	56.6 (17.36)
Baseline blood eosinophil count, cells/µL	
Geometric mean (SD logs)	290 (1.135)
≥150 cells/µL at screening, n (%)	125 (86)
≥300 cells/µL in previous 12 mo, n (%)	96 (66)
Omalizumab therapy prior to treatment^g^	
Duration, months, median (range)	29.6 (4, 161)
Frequency of dose, n (%)	
Every 2 wk	75 (52)
Monthly	69 (48)
Monthly dose, mg, median (range)^h^	450.0 (100, 1200)

Abbreviations: ACQ‐5, Asthma Control Questionnaire‐5; DPI, dry powder inhaler; ER, emergency room; FEV_1_, forced expiratory volume in 1 s; FVC, forced vital capacity; ICS, inhaled corticosteroid; ITT, intent‐to‐treat; LABA, long‐acting beta agonist; LAMA, long‐acting muscarinic antagonist; LTRA, leukotriene receptor antagonist; OCS, oral corticosteroid; SABA, short‐acting β_2_‐agonist; SC, subcutaneous; SCS, systemic corticosteroids; SD, standard deviation; SGRQ, St George's Respiratory Questionnaire.

a Adults (≥18 y) required ≥880 µg, adolescents (12‐17 y) required ≥440 µg fluticasone propionate per day.

b LABA was provided concomitant with ICS, as per standard of treatment guidelines.

c Exacerbations requiring treatment with SCS (intramuscular, intravenous, or oral) per protocol.

d Data not available for one patient.

e Scale scores: 0 = no impairment, 6 = maximum impairment.

f Scale scores: 0 = best possible health status, 100 = worst possible health status.

g Data for one patient who received omalizumab every 3 wk is not included.

h Additional patient who received alternating doses of 450/300 mg every 2 wk not included.

### 3.2 Primary endpoint

**Figure 2:**
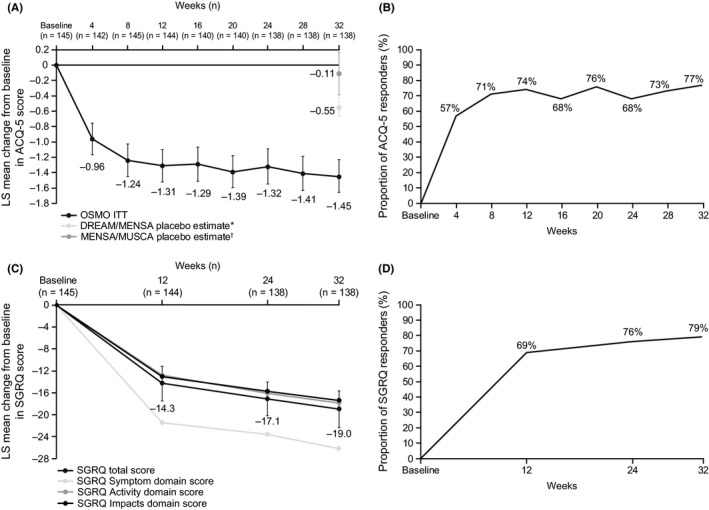
Analysis of (A) change from baseline in ACQ‐5 score, (B) the proportion of ACQ‐5 responders, (C) change from baseline in SGRQ total score, and (D) the proportion of SGRQ responders over the 32‐wk study period (ITT population). Vertical bars show 95% confidence intervals. *Placebo estimate of −0.55 (SE: 0.05), which was estimated from a meta‐analysis of studies MEA112997 (DREAM) and MEA115588 (MENSA) using all placebo patients; ^†^Placebo estimate of −0.11 (SE: 0.14), which was estimated from a meta‐analysis of studies MEA115588 (MENSA) and 200862 (MUSCA) using placebo patients who previously used omalizumab. ACQ‐5 responders were defined as patients with ≥0.5‐point reduction from baseline; SGRQ responders were defined as patients with ≥4‐point reduction from baseline; Analyses were performed using mixed model repeated measures with covariates of region, baseline maintenance OCS therapy (OCS, no OCS), exacerbations in the prior year, and visit. ACQ‐5, Asthma Control Questionnaire; ITT, intent‐to‐treat; LS, least squares; OCS, oral corticosteroid; SE, standard error; SGRQ, St George's Respiratory Questionnaire

Patient ACQ‐5 scores improved substantially over the study period, from a least squares (LS) mean score (SE) of 3.20 (0.076) at baseline to 1.75 (0.096) at Week 32 (Figure 2A), giving an LS mean change (SE) in ACQ‐5 score between baseline and Week 32 of −1.45 (0.107). Of the patients studied, 65/145 (45%) had an ACQ‐5 score <1.5 at Week 32, compared with 2/145 (1%) of patients at baseline. The 2 patients with an ACQ‐5 score <1.5 at baseline were enrolled in error after failing the ACQ‐5 continuation criterion (Table S1). The LS mean change from baseline at Week 32 in ACQ‐5 score was significantly improved compared with the DREAM/MENSA historical placebo control (difference: −0.90; 95% confidence intervals [CI] −1.13, −0.66; *P* < 0.001), and compared with the MENSA/MUSCA historical placebo control (difference: −1.34; 95% CI, −1.68, −1.00; *P* < 0.001). There was an early response in asthma control, with 103 patients (71%) achieving an improvement (reduction) in ACQ‐5 score of ≥0.5 points by Week 8. This improvement was maintained over the study period, with 111 patients (77%) meeting the MCID in ACQ‐5 score at Week 32 (Figure 2B).

### 3.3 Secondary endpoints

The LS mean (SE) SGRQ total score improved from 56.7 (1.36) at baseline to 37.8 (1.78) at Week 32, giving an LS mean change (SE) of −19.0 (1.64) (Figure 2C). The SGRQ domain with largest observed improvement was the SGRQ symptom domain score with an LS mean change (SE) of −26.2 (1.95) at Week 32. With regard to the percentage of SGRQ responders, 100 patients (69%) achieved an improvement (reduction) in SGRQ total score of ≥4 points by Week 12; this increased to 114 patients (79%) by Week 32 (Figure 2D).

Sixty patients (41%) experienced a total of 104 clinically significant exacerbations during the 32‐week study period, of whom 15 patients required an ER visit/hospitalization and 9 patients required hospitalization. During the study period, the annualized rates of clinically significant exacerbations and exacerbations requiring an ER visit/hospitalization were reduced by 64% and 69%, respectively, compared with the year prior to study enrollment (Table 2). The cumulative incidence for time to first clinically significant exacerbation is shown in Figure S1.

**Table 2:** Analysis of annualized rate of exacerbations^a^ with mepolizumab over the 32‐week study period (ITT population)

	Mepolizumab 100 mg SC N = 145
Clinically significant exacerbations	
Pretreatment^c^ annualized exacerbation rate	3.26
On‐treatment^d^ annualized exacerbation rate	1.18
Rate Ratio [On/Pretreatment] (95% CI)	0.36 (0.28, 0.47)^b^
Exacerbations requiring ER visit or hospitalization	
Pretreatment^c^ annualized exacerbation rate	0.63
On‐treatment^d^ annualized exacerbation rate	0.19
Rate ratio [On/Pretreatment] (95% CI)	0.31 (0.18, 0.53)^b^
Exacerbations requiring hospitalization	
Pretreatment^c^ annualized exacerbation rate	0.17
On‐treatment^d^ annualized exacerbation rate	0.12
Rate ratio [On/Pretreatment] (95% CI)	0.74 (0.40, 1.37)

Abbreviations: CI, confidence interval; ER, emergency room; GEE, generalized estimating equation; ITT, intent‐to‐treat; SC, subcutaneous; SCS, systemic corticosteroids.

a Performed using GEE model assuming a negative binomial distribution with a covariate of treatment period (pretreatment, 32‐wk study period), all exacerbations required treatment with SCS (intramuscular, intravenous, or oral) per protocol.

b Denotes *P* < 0.001.

c Pretreatment refers to the year prior to study enrollment.

d On‐treatment refers to the time between the first dose of mepolizumab and study conclusion, regardless of mepolizumab discontinuation.

In the subgroup of patients who experienced ≥2 exacerbations during the on‐treatment period (n = 24), the mean (SD) number of exacerbations during the 12 months prior to screening was 4.3 (3.61) compared with 3.3 (2.65) in the overall study population. Other disease characteristics were similar to those in the overall ITT population (Table S2). Despite experiencing ≥2 exacerbations during mepolizumab treatment, 6/24 (25%) patients in this subgroup still experienced a ≥10% reduction in exacerbation rate while on treatment compared with the 12 months prior to the study (Table S3).

**Figure 3:**
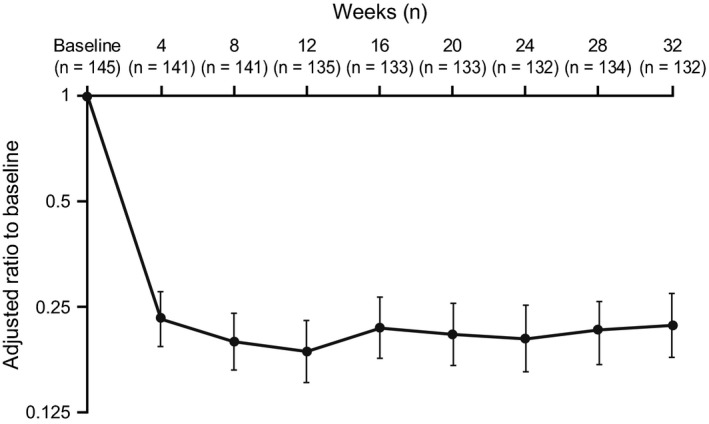
Blood eosinophil count adjusted ratio to baseline over the 32‐wk study period (ITT population). Vertical bars show 95% confidence intervals; Analyses were performed using mixed model repeated measures with covariates of region, baseline maintenance OCS therapy (OCS, no OCS), exacerbations in the prior year, and visit. ITT, intent‐to‐treat; OCS, oral corticosteroid

As observed in previous studies, blood eosinophil counts decreased rapidly from an LS mean (SE logs) of 290 cells/µL (0.091) at baseline to 70 cells/µL (0.073) at Week 4, and remained suppressed throughout the study period (LS mean [SE logs] at Week 32:60 cells/µL [0.081]). At Week 32, the ratio to baseline in blood eosinophil count was 0.22, demonstrating a 78% reduction from baseline (Figure 3). There was an associated fall in markers of eosinophil activity (Appendix S1).

### 3.4 Additional endpoints

Prebronchodilator FEV_1_ values increased over the treatment period from an LS mean (SE) of 1755 (56.7) mL at baseline to 1915 (63.3) mL at Week 32, giving an LS mean change (SE) from baseline at Week 32 of 159 (40.7) mL (Figure S2A). Similarly, postbronchodilator FEV_1_ values increased from an LS mean (SE) of 1987 (66.1) mL at baseline to 2106 (65.6)  mL at Week 32, giving an LS mean change (SE) from baseline of 120 (36.2) mL (Figure S2B). Both these results exceeded the MCID in FEV_1_ of 100 mL. Patient‐ and clinician‐rated responses to therapy supported the improvements seen in clinical outcomes (Appendix S1).

In subgroup analyses, improvements from baseline were seen in ACQ‐5 score, SGRQ total score, and prebronchodilator FEV_1_ at Week 32 both in patients who required maintenance OCS use at baseline and in those who did not (Table S4). Additionally, compared with the year prior to study enrollment, the annualized rate of clinically significant exacerbations was reduced during the study period by 51% in patients who required maintenance OCS use at baseline and by 69% in patients who did not (Table S4).

### 3.5 Safety

Overall, 124 patients (86%) experienced an on‐treatment AE (Table 3). The most frequently reported AEs were headache (28%) and viral upper respiratory tract infection (17%). Sixteen patients (11%) experienced an on‐treatment SAE, of whom 7 (5%) experienced asthma worsening. These seven patients did not display any common clinical characteristics, and all tested negative for ADAs. Overall, AEs were comparable in the first 16 weeks of the study, when omalizumab was within the patients’ system, and in the second 16 weeks of the study, when omalizumab is anticipated to have washed out (data not shown).

**Table 3:** Summary of on‐treatment^a^ adverse events (ITT population)

	Mepolizumab 100 mg SC N = 145
Any AE	124 (86)
Related to study treatment, as per investigator assessment	33 (23)
Leading to discontinuation of study treatment	2 (1)
ECG QT prolonged	1 (<1)
Urticaria	1 (<1)
Most common AEs (occurring in ≥10% of patients)	
Headache	41 (28)
Viral upper respiratory tract infection	24 (17)
Bronchitis	19 (13)
Arthralgia	14 (10)
Fatigue	14 (10)
Any SAE	16 (11)
Related to study treatment, as per investigator assessment	0
Leading to discontinuation of study treatment	0
Most common SAEs (occurring in >1 patient)	
Asthma	7 (5)
Cellulitis	2 (1)
Pneumonia	2 (1)
Fatal SAEs	0
Mepolizumab AESIs	
Systemic reactions	1 (<1)
Allergic/hypersensitivity reactions	1 (<1)
Nonallergic reactions	0
Anaphylaxis	0
Local injection site reactions	5 (3)
All infections^b^	87 (60)
Serious infections	6 (4)
Opportunistic infections	5 (3)
Malignancies	1 (<1)
Cardiac disorders^c^	7 (5)
Serious cardiac, vascular and thromboembolic events	3 (2)
Serious ischemic events	3 (2)

Abbreviations: AE, adverse event; AESI, adverse event of special interest; ECG, electrocardiogram; ITT, intent‐to‐treat; SAE, serious adverse event; SC, subcutaneous.

Data are presented as n (%).

a On‐treatment refers to the time between the first dose of mepolizumab and 4 wk after the last dose of mepolizumab.

b All infections include all events in Infections and infestations System Organ Class, most commonly viral upper respiratory tract infection (24/145 [17%]), bronchitis (19/145 [13%]), influenza (12/145 [8%]), and rhinitis (10/145 [7%]).

c Cardiac disorders include all events in Cardiac disorders System Organ Class.

Two patients (1%) experienced AEs that led to permanent treatment discontinuation: one patient reported a nonserious AE of worsening urticaria and subsequently re‐started omalizumab (which is indicated for the treatment of urticaria^7^); a second patient met the protocol defined ECG stopping criteria with nonserious AE of prolonged ECG QT. Neither AE was considered related to mepolizumab treatment by the investigator. Both patients discontinued treatment but remained within the study until completion.

No events of anaphylaxis were reported. One systemic hypersensitivity reaction was reported, which was described as a symptom of headache; this occurred in a patient 35 days after the first dose, and 4 days after the last dose, of mepolizumab. The reaction was assessed as mild and nonserious by the investigator and resolved with continued mepolizumab treatment. Five (3%) patients experienced local injection site reactions. All AESIs are summarized in Table 3.

At any time, postbaseline, eleven patients (8%) tested positive for ADAs, of whom one patient tested ADA‐positive prior to commencing mepolizumab treatment. All patients tested negative for neutralizing antibodies postbaseline.

## 4 DISCUSSION

Patients with severe eosinophilic asthma not optimally controlled by omalizumab experienced a clinically significant benefit in asthma control following a direct switch from omalizumab to mepolizumab. Over the study period, the adjusted LS mean change in ACQ‐5 score was substantially greater than the MCID of 0.5 points, demonstrating a significant improvement over two historical placebo control estimates. Furthermore, almost half of the study population (45%) achieved an ACQ‐5 score of <1.5 at Week 32. In addition, patients showed clinically significant improvements in lung function and health status, with 79% of patients experiencing a ≥4‐point improvement in SGRQ total score at Week 32. There was a substantial reduction in the rate of clinically significant exacerbations and exacerbations requiring ER visit/hospitalization. Additionally, analysis of the cumulative incidence of time to first exacerbation demonstrated that there was no increase in the risk of exacerbation over time as omalizumab was washed out.

All efficacy endpoints demonstrated an early response, with near‐maximal results being achieved within 8‐12 weeks. Improvements were maintained over the study period showing no evidence of additional benefit when both biologics were in the patients’ system, nor a decline in benefit as omalizumab washed out. In contrast, improvements in several endpoints continued to increase until Week 32. With clinical benefit being observed across a wide range of endpoints, there was no evidence of trade‐off between clinical outcomes. We acknowledge that although improvements in both pre‐ and postbronchodilator FEV_1_ exceeded the MCID of 100 mL, we may have expected greater improvements in FEV_1_ given the marked improvements seen in other parameters. This may be because FEV_1_, a directly measured value, may not reflect improvements in symptoms and asthma control that are perceived by patients with severe/refractory asthma, creating a dissociation between lung function, asthma symptom control and risk of exacerbations.^25^, ^26^, ^27^ It is also possible that patients with such severe and long‐standing asthma may have undergone remodeling, limiting their capacity to improve spirometry, a concept that is supported by the mean baseline postbronchodilator FEV_1_/FVC of 0.65 and FEV_1_ reversibility of 0.22 L. Despite switching patients from one biologic to another without a washout period, the safety and immunogenicity profiles of mepolizumab during this study were similar to previous clinical trials in patients with severe eosinophilic asthma.^28^

Omalizumab has previously been reported to reduce peripheral blood eosinophil counts in patients with severe asthma,^29^ and to have greater efficacy in terms of exacerbation reduction in patients with blood eosinophil counts ≥260 cells/µL^30^ or ≥300 cells/µL,^31^, ^32^ as compared with patients with lower blood eosinophil counts. Interestingly, the present study assessed patients whose asthma was unresponsive to a biologic treatment targeting the IgE pathway and who were switched to an alternative treatment targeting the IL‐5 pathway. The results showed a significant decrease in peripheral blood eosinophil count as early as 4 weeks after switching from omalizumab to mepolizumab, and an associated decrease in markers of eosinophil activity (eosinophilic cationic protein and eosinophil‐derived neurotoxin). This rapid decrease is consistent with previous studies of mepolizumab,^15^, ^16^, ^17^ and reflects the direct action and target engagement of mepolizumab on the eosinophil survival factor, IL‐5. This study has demonstrated that mepolizumab is a relevant treatment option in patients with severe eosinophilic asthma that is unresponsive to high‐dose ICS and omalizumab, and that physicians may substitute one biologic with another to improve clinical outcomes.

The design of this study reflected expected clinical practice where mepolizumab would be started 2‐4 weeks after the final dose of omalizumab. Although we did not assess potential interaction between the two biologics, there was no evidence of negative interactions in terms of tolerability. Similarly, there was no evidence of greater efficacy during the first half of the mepolizumab treatment period as compared to the second half, suggesting that there was no positive interaction during the potential washout period from omalizumab. Patients in the ITT population had baseline ACQ‐5 scores, and other disease parameters that were representative of severe asthma and poor asthma control. Of note, the worse (higher) ACQ‐5 and SGRQ baseline scores allowed greater room for improvement over the study period. In addition, the average body mass index (BMI) of patients included in this study was 30.2 kg/m^2^ (range: 18.7‐48.4 kg/m^2^). Obesity, defined as BMI > 30.0 kg/m^2^, has been repeatedly associated with increased asthma severity and decreased response to glucocorticoid‐based therapies.^33^, ^34^, ^35^ It is possible that patients in the ITT population were experiencing obesity‐related asthma, which may have contributed to the severity of their disease and lack of response to standard of care therapies. Notwithstanding, the majority (77%) of patients in the ITT population experienced a clinically significant improvement in asthma control in response to mepolizumab.

The principal limitation of this study was the single‐arm design without a randomized control group. This limitation was partly addressed using historical placebo control estimates, generated by meta‐analyses of previous clinical trials of mepolizumab in patients with severe eosinophilic asthma. The clinical trials used in these meta‐analyses were of similar length and had comparable inclusion and exclusion criteria; therefore, the “placebo estimates” were based on patients of similar clinical status and disposition. In particular, the meta‐analysis of MENSA/MUSCA only included patients who had previously received omalizumab therapy. Secondly, this was a 32‐week, rather than 12‐month study. Thirty‐two weeks is shorter than the ideal time frame for assessment of exacerbation rates; however, this limitation was partly mitigated through recruitment of patients across seasons, reducing seasonality confounding. Furthermore, although clinical conditions may vary when comparing exacerbation rates pre‐ and poststudy, objective measures such as FEV_1_ and blood eosinophil counts showed the same trend as exacerbation rates over this time frame. Thirdly, as patients were receiving omalizumab treatment prior to entering the study, the initial indications for prescribing omalizumab were not known for all patients, and therefore could have been incorrect in a subset. Nonetheless, it is thought that these patients were likely to have been identified as suitable for omalizumab treatment according to the product label and, as such, would have been diagnosed with allergic asthma and received the appropriate dose of omalizumab based on their IgE levels. Indeed, one of the study inclusion criteria stated that study patients must have been receiving omalizumab based on weight and IgE levels. As patients were not required to washout prior to the switch to mepolizumab, baseline measurements of atopy were not collected as these would have been confounded by current omalizumab treatment. Finally, to justify the study treatment, this study was conducted in a subgroup of patients eligible for both omalizumab and mepolizumab who had uncontrolled disease. Consequently, the results of this study are not necessarily generalizable to the entire overlapping population of patients eligible for both biologics.

In conclusion, this open‐label study provides evidence that patients with severe asthma who are eligible for both biologics and not optimally controlled with omalizumab could be effectively switched to mepolizumab to improve their asthma control. Patients experienced statistically significant and clinically meaningful improvements in asthma control (measured by ACQ‐5), quality of life (measured by SGRQ), and asthma exacerbations, following the switch from omalizumab to mepolizumab without a standard washout. No tolerability issues were observed. This pragmatic study reflects expected clinical practice and provides practical guidance to clinicians for the treatment of patients with severe eosinophilic asthma.

## CONFLICTS OF INTEREST

FCA, JA, DM, DG, and RGP are all employees of GSK and hold stocks/shares in GSK. KRC has received consulting fees from AstraZeneca, Boehringer Ingelheim, CSL Behring, GSK, Grifols, Kamada, Novartis, Roche, and Sanofi Regeneron; has undertaken research funded by Amgen, AstraZeneca, Boehringer Ingelheim, CSL Behring, GSK, Grifols, Kamada, Novartis, Roche and Sanofi; has participated in continuing medical education activities sponsored in whole or in part by AstraZeneca, Boehringer Ingelheim, GSK, Grifols, Novartis, and Teva. KRC is participating in research funded by the Canadian Institutes of Health Research and holds the GSK‐CIHR Research Chair in Respiratory Health Care Delivery at the University Health Network, Toronto, Canada. GD has received personal fees or research grants from Novartis Pharma, AstraZeneca, GSK, Boehringer Ingelheim, Mundi Pharma, Vivisol, Sanofi, Chiesi, ALK, TEVA, MSD, AGIR à Dom; has participated in CME activities sponsored by GSK, AstraZeneca, Novartis Pharma, Chiesi, MSD, Takeda, AGIR à Dom, Orkyn, Mundi Pharma, ALK, Stallergenes, Boehringer Ingelheim, TEVA; is participating in research funded by GSK, ALK, AstraZeneca, Novartis Pharma, Boehringer Ingelheim, Vitalair, AB science, Amgen, Lilly, Sanofi, Roche, TEVA. MB has received personal fees or research grants from AstraZeneca, Novartis, GSK, Boehringer Ingelheim, and Sanofi. MCL has received research grants or personal fees from Boehringer Ingelheim, GSK, Mereo Biopharm, and MedImmune. BC is on the speaker's Bureau and advisor for consult for the following companies: AstraZeneca, Boehringer Ingelheim, Circassia, Genentech, Novartis, Teva. XM has received fees as a speaker, scientific advisor or participant of clinical studies for AstraZeneca, Boehringer Ingelheim, Chiesi, Faes, GlaxoSmithKline, Menarini, Mundifarma, Novartis and Teva.

## AUTHOR CONTRIBUTIONS

FCA, DM, RGP, and DG contributed to the conception and design of the study. MCL, BC, KRC, XM, GD, and MB contributed to the acquisition of data. All authors were involved in data analysis and interpretation, development of the manuscript and approval of the final draft to be published.

## DATA SHARING

Anonymized individual participant data and study documents can be requested for further research from http://www.clinicalstudydatarequest.com.

## References

[1] WHO . Asthma fact sheet. 2017; http://www.who.int/mediacentre/factsheets/fs307/en/. Accessed January 10, 2018.

[2] Chung KF , Wenzel SE , Brozek JL , et al. International ERS/ATS guidelines on definition, evaluation and treatment of severe asthma. Eur Respir J. 2014;43(2):343‐373. 24337046 10.1183/09031936.00202013

[3] Soriano JB , Abajobir AA , Abate KH , et al. Global, regional, and national deaths, prevalence, disability‐adjusted life years, and years lived with disability for chronic obstructive pulmonary disease and asthma, 1990–2015: a systematic analysis for the Global Burden of Disease Study 2015. Lancet Respir Med. 2017;5(9):691‐706. 28822787 10.1016/S2213-2600(17)30293-X PMC5573769

[4] Al Efraij K , FitzGerald JM . Current and emerging treatments for severe asthma. J Thorac Dis. 2015;7(11):E522‐E225. 26716048 10.3978/j.issn.2072-1439.2015.10.73 PMC4669299

[5] Wenzel S . Severe asthma: from characteristics to phenotypes to endotypes. Clin Exp Allergy. 2012;42(5):650‐658. 22251060 10.1111/j.1365-2222.2011.03929.x

[6] Tran TN , Zeiger RS , Peters SP , et al. Overlap of atopic, eosinophilic, and TH2‐high asthma phenotypes in a general population with current asthma. Ann Allergy Asthma Immunol. 2016;116(1):37‐42. 26707771 10.1016/j.anai.2015.10.027

[7] FDA . Xolair (omalizumab) prescribing information. 2003; https://www.accessdata.fda.gov/drugsatfda_docs/label/2016/103976s5225lbl.pdf. Accessed December 1, 2017.

[8] MacGlashan DW Jr , Bochner BS , Adelman DC , et al. Down‐regulation of Fc(epsilon)RI expression on human basophils during in vivo treatment of atopic patients with anti‐IgE antibody. J Immunol. 1997;158(3):1438‐1445. 9013989

[9] Hanania NA , Alpan O , Hamilos DL , et al. Omalizumab in severe allergic asthma inadequately controlled with standard therapy: a randomized trial. Ann Intern Med. 2011;154(9):573‐582. 21536936 10.7326/0003-4819-154-9-201105030-00002

[10] Holgate ST , Chuchalin AG , Hebert J , et al. Efficacy and safety of a recombinant anti‐immunoglobulin E antibody (omalizumab) in severe allergic asthma. Clin Exp Allergy. 2004;34(4):632‐638. 15080818 10.1111/j.1365-2222.2004.1916.x

[11] Humbert M , Beasley R , Ayres J , et al. Benefits of omalizumab as add‐on therapy in patients with severe persistent asthma who are inadequately controlled despite best available therapy (GINA 2002 step 4 treatment): INNOVATE. Allergy 2005;60(3):309‐316. 15679715 10.1111/j.1398-9995.2004.00772.x

[12] GINA . Global strategy for asthma management and prevention. 2017; http://ginasthma.org/2017-gina-report-global-strategy-for-asthma-management-and-prevention/. Accessed January 10, 2018.

[13] FDA . Nucala (mepolizumab) prescribing information. 2015; https://www.accessdata.fda.gov/drugsatfda_docs/label/2015/125526Orig1s000Lbl.pdf. Accessed December 1, 2017.

[14] Mukherjee M , Sehmi R , Nair P . Anti‐IL5 therapy for asthma and beyond. World Allergy Organ J. 2014;7(1):32. 25709744 10.1186/1939-4551-7-32 PMC4326373

[15] Bel EH , Wenzel SE , Thompson PJ , et al. Oral glucocorticoid‐sparing effect of mepolizumab in eosinophilic asthma. N Engl J Med. 2014;371(13):1189‐1197. 25199060 10.1056/NEJMoa1403291

[16] Ortega HG , Liu MC , Pavord ID , et al. Mepolizumab treatment in patients with severe eosinophilic asthma. N Engl J Med. 2014;371(13):1198‐1207. 25199059 10.1056/NEJMoa1403290

[17] Pavord ID , Korn S , Howarth P , et al. Mepolizumab for severe eosinophilic asthma (DREAM): a multicentre, double‐blind, placebo‐controlled trial. Lancet 2012;380(9842):651‐659. 22901886 10.1016/S0140-6736(12)60988-X

[18] Chupp GL , Bradford ES , Albers FC , et al. Efficacy of mepolizumab add‐on therapy on health‐related quality of life and markers of asthma control in severe eosinophilic asthma (MUSCA): a randomised, double‐blind, placebo‐controlled, parallel‐group, multicentre, phase 3b trial. Lancet Respir Med. 2017;5(5):390‐400. 28395936 10.1016/S2213-2600(17)30125-X

[19] Magnan A , Bourdin A , Prazma CM , et al. Treatment response with mepolizumab in severe eosinophilic asthma patients with previous omalizumab treatment. Allergy 2016;71(9):1335‐1344. 27087007 10.1111/all.12914 PMC5089585

[20] GINA . Global strategy for asthma management and prevention. 2015; http://ginasthma.org/wp-content/uploads/2016/01/GINA_Report_2015_Aug11-1.pdf.

[21] NHLBI . Exper panel report 3: guideines for the diagnosis and management of asthma. National asthma education and prevention program. 2007; https://www.nhlbi.nih.gov/files/docs/guidelines/asthgdln.pdf

[22] Juniper EF , Svensson K , Mork AC , Stahl E . Measurement properties and interpretation of three shortened versions of the asthma control questionnaire. Respir Med. 2005;99(5):553‐558. 15823451 10.1016/j.rmed.2004.10.008

[23] Jones PW . Interpreting thresholds for a clinically significant change in health status in asthma and COPD. Eur Respir J. 2002;19(3):398‐404. 11936514 10.1183/09031936.02.00063702

[24] Keene ON , Davis RL , Koch GG . Use of generalized estimating equations in a trial in influenza to explore treatment effects over time. Pharm Stat. 2004;3(4):281‐287.

[25] Green RH , Brightling CE , McKenna S , et al. Asthma exacerbations and sputum eosinophil counts: a randomised controlled trial. Lancet 2002;360(9347):1715‐1721. 12480423 10.1016/S0140-6736(02)11679-5

[26] Jayaram L , Pizzichini MM , Cook RJ , et al. Determining asthma treatment by monitoring sputum cell counts: effect on exacerbations. Eur Respir J. 2006;27(3):483‐494. 16507847 10.1183/09031936.06.00137704

[27] Haldar P , Pavord ID , Shaw DE , et al. Cluster analysis and clinical asthma phenotypes. Am J Respir Crit Care Med. 2008;178(3):218‐224. 18480428 10.1164/rccm.200711-1754OC PMC3992366

[28] Lugogo N , Domingo C , Chanez P , et al. Efficacy and safety of mepolizumab in patients with severe eosinophilic asthma: a multi‐center, open‐label, phase IIIb study. Clin Ther. 2016;38(9):2058–2070. 27553751 10.1016/j.clinthera.2016.07.010

[29] Massanari M , Holgate ST , Busse WW , Jimenez P , Kianifard F , Zeldin R . Effect of omalizumab on peripheral blood eosinophilia in allergic asthma. Respir Med. 2010;104(2):188–196. 19846286 10.1016/j.rmed.2009.09.011

[30] Hanania NA , Wenzel S , Rosén K , et al. Exploring the effects of omalizumab in allergic asthma: an analysis of biomarkers in the EXTRA study. Am J Respir Crit Care Med. 2013;187(8):804–811. 23471469 10.1164/rccm.201208-1414OC

[31] Busse W , Spector S , Rosen K , Wang Y , Alpan O . High eosinophil count: a potential biomarker for assessing successful omalizumab treatment effects. J Allergy Clin Immunol. 2013;132(2):485–486. 23591271 10.1016/j.jaci.2013.02.032

[32] Manga V , Humbert M , Djukanovic R , et al. Eosinophils and serum IgE predict response to omalizumab in patients with severe allergic asthma: innovate trial post‐hoc analysis. J Allergy Clin Immunol. 2016;137(2):AB16.

[33] Akerman MJ , Calacanis CM , Madsen MK . Relationship between asthma severity and obesity. J Asthma. 2004;41(5):521–526. 15360059 10.1081/jas-120037651

[34] Peters‐Golden M , Swern A , Bird SS , Hustad CM , Grant E , Edelman JM . Influence of body mass index on the response to asthma controller agents. Eur Respir J. 2006;27(3):495–503. 16507848 10.1183/09031936.06.00077205

[35] Sutherland ER , Lehman EB , Teodorescu M , Wechsler ME , National Heart L , Blood Institute's Asthma Clinical Research N . Body mass index and phenotype in subjects with mild‐to‐moderate persistent asthma. J Allergy Clin Immunol. 2009;123(6):1328–1334. 19501235 10.1016/j.jaci.2009.04.005 PMC2743451

