# Supplementary material for: The clinical benefit of mepolizumab replacing omalizumab in uncontrolled severe eosinophilic asthma
Source: Allergy. 2019 Jul 1;74(9):1716–26. doi: 10.1111/all.13850 (PMC6790683; doi:10.1111/all.13850)
Supplement: Supplementary file 3 [file ALL-74-1716-s003.docx]

**Supplementary materials**

**The clinical benefit of mepolizumab replacing omalizumab in uncontrolled severe eosinophilic asthma (Chapman *et al*.)**

**Contents**

| *Supplementary methods* |
| --- |
| Study locations |
| Clinically significant exacerbations |
| Inclusion criteria |
| Exclusion criteria |
| Inflammatory biomarkers |
| Adverse events of special interest |
| *Supplementary results* |
| Inflammatory biomarkers |
| Clinician and patient-reported outcomes |
| *Supplementary tables* |
| Supplementary Table 1. Summary of inclusion, exclusion and continuation criteria failed by patients included in the ITT population |
| Supplementary Table 2. Baseline asthma characteristics in patients who experienced ≥2 exacerbations in the on-treatment period. |
| Supplementary Table 3. Reduction in on-treatment exacerbation rate compared to 12-months prior to study enrollment. |
| Supplementary Table 4. Analysis of clinical endpoints with mepolizumab over the 32-week study period, according to baseline maintenance OCS use (ITT population). |
| *Supplementary figures* |
| Figure S1. Kaplan–Meier cumulative incidence curve for time to first clinically significant exacerbation (ITT population). |
| Figure S2. Analysis of change from baseline in (A) pre-bronchodilator FEV_1,_ and (B) post-bronchodilator FEV_1_, over the 32-week study period (ITT population). |

**Supplementary Material**

**Methods**

*Study locations*

This study was conducted at 46 centers: six in Argentina, one in Belgium, eight in Canada, eight in

France, four in Germany, two in the Netherlands, six in Spain, two in Sweden and nine in the United

States.

*Clinically significant exacerbation*

A clinically significant exacerbation was defined as a worsening of asthma which requires treatment with systemic corticosteroids, an emergency room visit or hospitalization. Systemic corticosteroids had to be intravenous or oral steroids for at least 3 days, or a single intramuscular dose. For patients on maintenance systemic corticosteroids, at least double the existing dose for at least 3 days was required.

*Inclusion criteria*

At Visit 1, patients were required to have a pre-bronchodilator forced expiratory volume in 1 second (FEV_1_) of <80% of the predicted value (if ≥18 years of age), a pre-bronchodilator FEV_1_ <90% of the predicted value (if 12–17 years of age), or a ratio of FEV_1_ to forced vital capacity (FVC) of <0.8.

High dose inhaled corticosteroid (ICS) was defined as ≥880 mcg/day fluticasone propionate (for patients ≥18 years of age) or ≥440 mcg/day fluticasone propionate (for patients 12–17 years of age); for ICS/LABA combinations the highest approved maintenance dose in the local country met this criterion.

*Exclusion criteria*

Patients were excluded from the study if they had a concurrent medical condition or disease, including: clinically important lung disease other than asthma; malignancy or a history of cancer in the past 12 months; liver disease, defined as an active liver or biliary disease or alanine aminotransferase >2x upper limit of normal (ULN) and bilirubin >1.5xULN at Visit 1; chronic hepatitis B; cardiovascular disease uncontrolled with standard treatment; any patient with QTc >450 msec (or >480 msec in patients with bundle branch block) at Visit 1; any other eosinophilic disease; immunodeficiency. Patients were also excluded if they had received any monoclonal antibody therapy, other than omalizumab, to treat inflammatory disease within 5 half-lives of Visit 1, if they were a current or former smoker with a history of ≥10 pack years, or if they had a history of alcohol or substance abuse.

*Inflammatory biomarkers*

Levels of eosinophilic cationic protein (ECP), eosinophil-derived neurotoxin (EDN) and total interleukin (IL)-5 were assessed at baseline, Week 8 and Week 32. ECP was assessed by means of an ImmunoCAP ECP assay; EDN was assessed using an Imunodiagnostik EDN ELISA assay; total IL-5 was assessed using a validated MSD immunoassay, details of which have been reported previously.^1^ All inflammatory biomarkers were summarised using descriptive statistics.

*Adverse events of special interest*

Within the mepolizumab clinical development program AESIs include: systemic (allergic/hypersensitivity and non-allergic) reactions, local injection site reactions, infections (including serious and opportunistic infections), malignancies, serious cardiac, vascular and thromboembolic events and serious ischemic events.

**Results**

*Inflammatory biomarkers*

At Week 32, ECP levels ranged from below the limit of quantification (BLQ) to 36.00 µg/L, and EDN levels ranged from BLQ to 2007.00 µg/L. The ratio to baseline at Week 32 for levels of ECP and EDN were 0.47 and 0.33, respectively. There was also an accumulation of total IL-5 level, with the total IL-5 level at Week 32 ranging from BLQ to 1053.30 ng/L, and a ratio to baseline at Week 32 of 14.34.

*Clinician and patient-reported outcomes*

The majority of patients (85%) and clinicians (86%) rated the overall response to therapy as mildly, moderately or significantly improved. In addition, at Week 32, there was a mean change (SD) from baseline in patients’ Treatment Satisfaction Questionnaire for Medication-9 Overall Satisfaction Scale scores of 17 (34.8) points (p<0.001).

**Supplementary Table 1. Summary of inclusion, exclusion and continuation criteria failed by patients included in the ITT population**

|  | **Mepolizumab 100 mg SC**  **(N=145)** |
| --- | --- |
| **Any criteria failed** | **14 (10)** |
| **Inclusion criteria failed** | |
| Eosinophilic asthma: peripheral blood eosinophil count ≥300 cells/µL in the 12 months prior to Visit 1 or ≥150 cells/µL at Visit 1. | 2 (1) |
| ICS: Regular treatment with high-dose ICS in the 12 months prior to Visit 1 with or without maintenance OCS. Patients ≥18 years of age: ICS ≥880 mcg. Patients 12–17 years of age: ICS ≥440 mcg. If receiving ICS/LABA combination therapy, the highest ICS dose in local country meets this criterion. | 3 (2) |
| Controller medication: Treatment with an additional controller besides ICS for ≥3 months or documented failure in the past 12 months of an additional controller for ≥3 successive months. | 1 (<1) |
| Asthma symptoms not optimally controlled: An ACQ-5 score of ≥1.5 recorded at  Visit 1. | 1 (<1) |
| Exacerbation history: History of ≥2 exacerbations in the 12 months prior Visit 1 despite high-dose ICS. If on omalizumab for ≥8 months, at least 1 exacerbation to have occurred while receiving omalizumab. | 2 (1) |
| Sex: Male or female patient of non-child bearing potential or female patients of child bearing potential with negative pregnancy test at Visit 1 agreeing to follow an acceptable contraceptive method. | 1 (<1) |
| **Exclusion criteria failed** | |
| ECG: Patients with QTc > 450 msec or QTc > 480 msec in patients with bundle branch block at Visit 1. | 1 (<1) |
| **Continuation criteria failed** | |
| Not optimally controlled: An ACQ-5 score of ≥1.5 recorded at Visit 2. | 2 (1) |
| Last dose of omalizumab: Patients receiving omalizumab every 4 weeks: last dose must be 3–5 weeks prior to Visit 2. Patients receiving omalizumab every 2 weeks: last dose must be 1–3 weeks prior to Visit 2. | 5 (3) |

Data are presented as n (%). Patients can have failure with more than 1 inclusion/exclusion/continuation criterion.

ACQ-5, Asthma Control Questionnaire-5; ECG, electrocardiogram; ICS, inhaled corticosteroid; ITT, intent-to-treat; LABA; long-acting β_2_-agonist; OCS, oral corticosteroid; SC, subcutaneous

**Supplementary Table 2. Baseline asthma characteristics in patients who experienced ≥2 exacerbations in the on-treatment period.**

| **Characteristic** | **Mepolizumab 100 mg SC**  **(N=24)** |
| --- | --- |
| Duration of asthma, years, mean (SD) | 26.4 (19.19) |
| Maintenance OCS use at baseline, n (%)  Median (range) dose, mg/day prednisolone equivalent | 8 (33)  17.5 (5, 30) |
| Exacerbations* in the past 12 months |  |
| Clinically significant exacerbations, mean (SD) | 4.3 (3.61) |
| Exacerbations requiring ER/hospitalization, n (%) | 11 (46) |
| Exacerbations requiring hospitalization, n (%) | 6 (25) |
| Baseline blood eosinophil count, cells/µL |  |
| ≥150 cells/µL at screening, n (%) | 20 (83) |
| ≥300 cells/µL in previous 12 months, n (%) | 16 (67) |

*Exacerbations requiring treatment with systemic corticosteroids (intramuscular, intravenous or oral) per protocol

OCS, oral corticosteroid; SC, subcutaneous; SD, standard deviation

**Supplementary Table 3. Reduction in on-treatment exacerbation rate compared to 12-months prior to study enrollment.**

|  | **Overall ITT population**  **(N=145)** | **Patients who experienced ≥2 exacerbations during the on-treatment period**  **(N=24)** |
| --- | --- | --- |
| **Patients experiencing reduction in on-treatment exacerbation rate compared to 12-months prior to study enrollment, n (%)**  ≥10% reduction  ≥20% reduction  ≥30% reduction  ≥40% reduction  ≥50% reduction | 125 (86)  116 (80)  109 (75)  109 (75)  100 (69) | 6 (25)  4 (17)  3 (13)  3 (13)  3 (13) |

ITT, intent-to-treat.

**Supplementary Table 4. Analysis of clinical endpoints with mepolizumab over the 32-week study period, according to baseline maintenance OCS use (ITT population).**

|  | **Mepolizumab 100 mg SC**  **(N=145)** | |
| --- | --- | --- |
|  | **Baseline maintenance OCS use: No**  **(N=110)** | **Baseline maintenance OCS use: Yes**  **(N=35)** |
| ACQ-5 score^¶^ |  |  |
| LS mean (SE) at baseline | 3.07 (0.088) | 3.60 (0.157) |
| LS mean (SE) at Week 32 | 1.65 (0.111) | 2.08 (0.195) |
| LS mean (SE) change from baseline to Week 32 | -1.41 (0.135) | -1.52 (0.144) |
| SGRQ total score^¶^ |  |  |
| LS mean (SE) at baseline | 54.4 (1.65) | 63.7 (2.24) |
| LS mean (SE) at Week 32 | 35.8 (2.02) | 43.7 (3.57) |
| LS mean (SE) change from baseline to Week 32 | -18.7 (2.03) | -20.0 (2.39) |
| Clinically significant exacerbations* |  |  |
| Pre-treatment^†^ annualized exacerbation rate | 3.09 | 3.77 |
| On-treatment^‡^ annualized exacerbation rate | 0.95 | 1.85 |
| Rate Ratio [On/Pre-treatment] (95% CI) | 0.31 (0.23, 0.42)^§^ | 0.49 (0.30, 0.81)^║^ |
| Pre-bronchodilator FEV_1_^¶^ |  |  |
| LS mean (SE) at baseline, L | 1.8 (0.66) | 1.6 (0.11) |
| LS mean (SE) at Week 32, L | 2.0 (0.72) | 1.7 (0.13) |
| LS mean (SE) change from baseline to Week 32, mL | 207 (44.1) | 15 (92.8) |

^¶^Analyses were performed using mixed model repeated measures with covariates of region, baseline maintenance OCS therapy (OCS, no OCS), exacerbations in the prior year, and visit. ^*^Performed using GEE model assuming a negative binomial distribution with a covariate of treatment period (pre-treatment, 32 week study period), all exacerbations required treatment with systemic corticosteroids (intramuscular, intravenous or oral) per protocol; ^†^Pre-treatment refers to the year prior to study enrollment; ^‡^On-treatment refers to the time between the first dose of mepolizumab and study conclusion, regardless of mepolizumab discontinuation; ^§^denotes p<0.001; ^║^denotes p=0.005.

ACQ, Asthma Control Questionnaire; CI, confidence interval; FEV_1_, forced expiratory volume in 1 second; GEE, generalized estimating equation; ITT, intent-to-treat; LS, least squares; OCS, oral corticosteroids; SC, subcutaneous; SE, standard error; SGRQ, St George’s Respiratory Questionnaire.

**SUPPLEMENTARY FIGURE LEGENDS**

**Figure S1.** Kaplan–Meier cumulative incidence curve for time to first clinically significant exacerbation (ITT population).


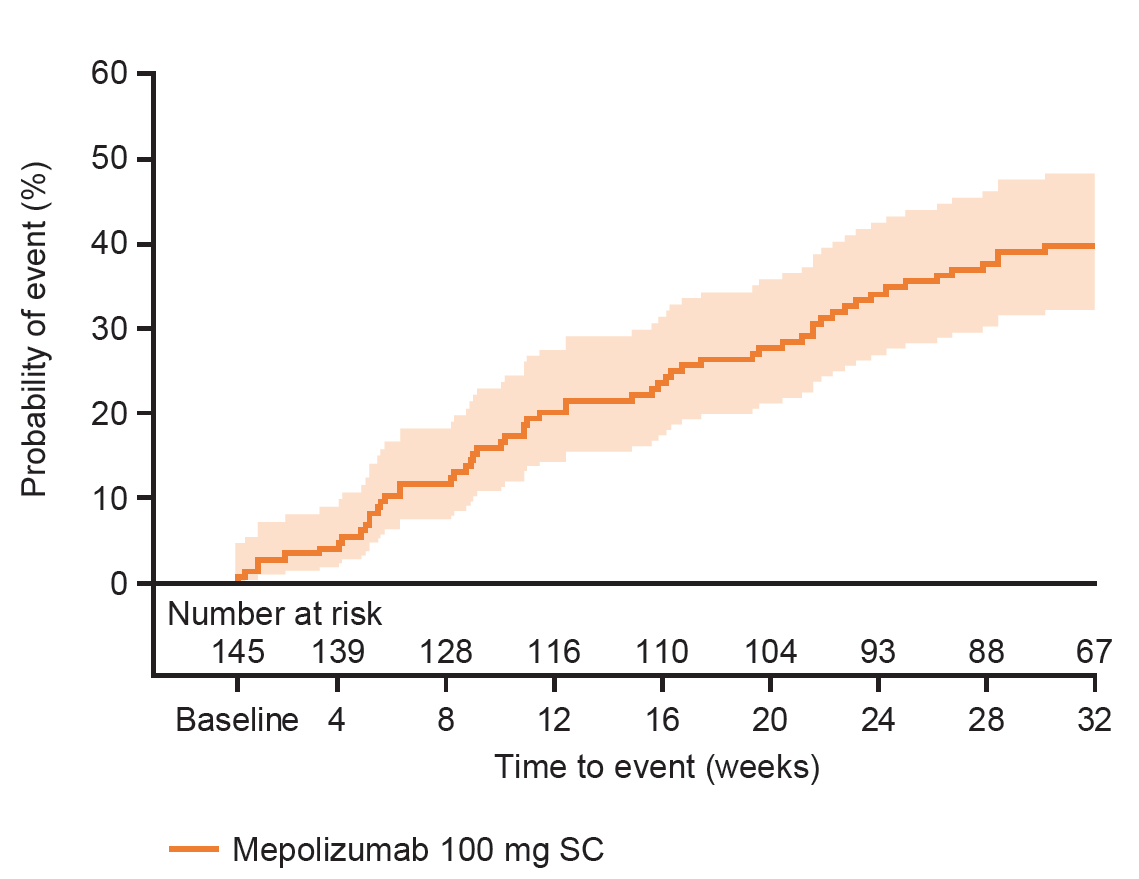


Shaded areas represent 95% confidence interval

ITT, intent intent-to-treat; SC, subcutaneous.

**Figure S2.** Analysis of change from baseline in (A) pre-bronchodilator FEV_1,_ and (B) post-bronchodilator FEV_1_, over the 32-week study period (ITT population).


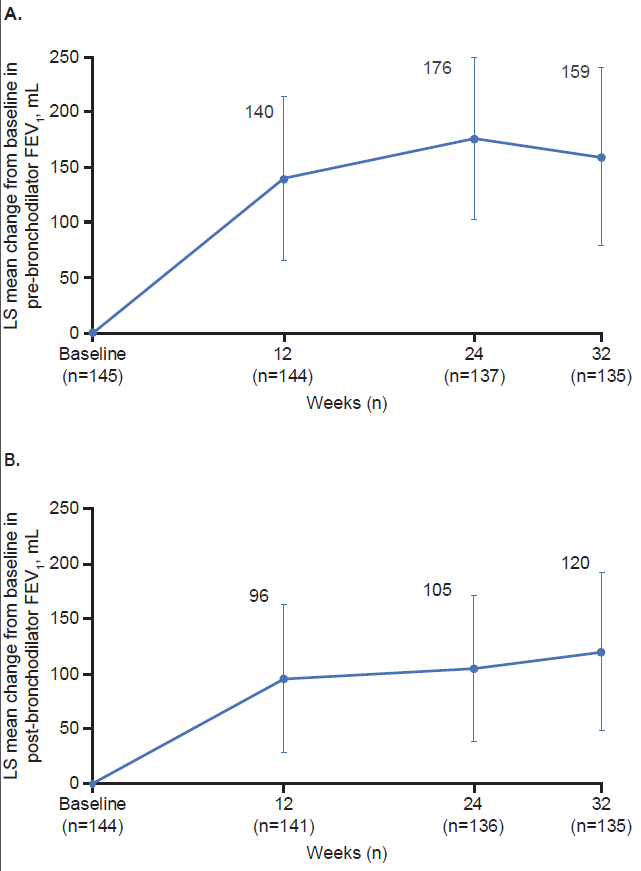


Vertical bars show 95% confidence intervals. Minimal clinically important difference: 100 mL improvement

Analyses were performed using mixed model repeated measures with covariates of region, baseline maintenance OCS therapy (OCS, no OCS), exacerbations in the prior year, and visit.

FEV_1_, forced expiratory volume in 1 second; ITT, intent-to-treat; LS, least squares; mL, milliliters; OCS, oral corticosteroid.

**References**

1. Liao K, Meyer E, Lee TN, Loercher A, Sikkema D. Inhibition of interleukin-5 induced false positive anti-drug antibody responses against mepolizumab through the use of a competitive blocking antibody. *J Immunol Methods* 2017; **441**: 15-23.
